# Whole genome sequencing reveals genomic diversity and evidence of hybridization in Moroccan *Leishmania infantum* strains

**DOI:** 10.3389/fcimb.2026.1872182

**Published:** 2026-07-09

**Authors:** Sara El Mazini, Hasnaa Talimi, Pascale Pescher, Delphine Bacq-Daian, Robert Olaso, Jean François Deleuze, Khadija Bekhti, Gerald F. Späth, Meryem Lemrani

**Affiliations:** 1 Laboratory of Parasitology and Vector-Borne-Diseases, Institut Pasteur du Maroc, Casablanca, Morocco; 2 Laboratory of Microbial Biotechnology and Bioactive Molecules, Faculty of Sciences and Technologies, Sidi Mohammed Ben Abdellah University, Fes, Morocco; 3 Institut Pasteur, Université Paris Cité, INSERM U1347, Unité de Parasitologie moléculaire et Signalisation, Paris, France; 4 Centre National de Recherche en Génomique Humaine (CNRGH), Institut de Biologie François Jacob, Commissariat à l'énergie atomique et aux énergies alternatives (CEA), Université Paris-Saclay, Evry, France

**Keywords:** *Leishmania infantum*, CL/HIV, genomic diversity, aneuploidy, CNV, SNP, hybridization, Morocco

## Abstract

In Morocco, *Leishmania infantum* is an endemic species responsible for visceral leishmaniasis (VL, fatal if untreated) as well as cutaneous leishmaniasis (CL). The number of cases and the distribution range of both forms are increasing, spreading into new areas. However, despite its clinical significance, the genomic diversity of this species remains largely overlooked. We performed whole-genome sequencing of six *L. infantum* strains from patients with VL, CL, or CL/HIV, as well as from dogs, and conducted comparative genomic analyses. Read depth analysis at both the chromosome and gene levels revealed isolate-specific variability in ploidy and identified gene copy number variations (CNVs), mostly in genes involved in parasite virulence and survival, all of which support the parasite’s remarkable adaptability. Principal component analysis (PCA) and SNP-based clustering revealed a notable divergence between the two cutaneous isolates and the other strains. We further confirmed the diversity of the CL/HIV strain previously studied using the MLST technique. In addition, the pronounced genomic heterogeneity observed in this strain suggests that host immune status may influence parasite diversity. The strain FA23, isolated from a CL patient exhibiting substantial SNP divergence from all other isolates, suggested a possible hybrid origin, which we confirmed using the Kraken2 package, which revealed important introgression with genome sequences from *L. donovani* (22.21%). These findings demonstrate that comparative genomics are instrumental in detecting hybridization signals, supporting genetic exchange as a significant evolutionary process contributing in the diversification of new CL cases caused by *L. infantum*.

## 1 Introduction

*Leishmania infantum* is one of the main agents responsible for leishmaniasis in the Mediterranean basin, including Morocco. It causes both visceral and cutaneous forms of the disease. Zoonotic visceral leishmaniasis, caused by *L. infantum* MON-1, has been present in Morocco since 1920 (Hmamouch et al., 2014), with a particularly high prevalence in the north of the country (Taounate) (Rhajaoui, 2011) and sporadic cases reported in the south (Marrakech) (Kahime et al., 2017). *L. infantum* is also responsible for cutaneous leishmaniasis, the first case having been reported in 1996 in the province of Taounate, where a MON-24 dermotropic variant was identified (Rioux et al., 1996; Lemrani et al., 2002).

In Morocco, seven regions have been identified as high-risk areas for HIV/leishmaniasis co-infection: Marrakech-Safi, Casablanca-Settat, Rabat-Salé-Kénitra, Fès-Meknès, Tanger-Tétouan-Al Hoceima, Oriental, and Souss-Massa. Among these high-risk regions, anti-*Leishmania infantum* antibodies were detected in 5% of HIV-positive patients in Marrakech-Safi (Echchakery et al., 2018). Most deaths associated with HIV/leishmaniasis co-infection are attributable to visceral leishmaniasis. In contrast, HIV/cutaneous leishmaniasis co-infection is less frequently reported. Nevertheless, considerable clinical variability has been observed in several cutaneous leishmaniasis foci in northern and central Morocco, suggesting the possibility of co-infection. A case of HIV/cutaneous leishmaniasis co-infection was reported in the Souss-Massa region (Ouadi et al., 2016).

In Morocco, several studies have investigated and demonstrated the genetic diversity of *L. infantum* using various techniques and markers, including multilocus microsatellite typing (MLMT) (Amro et al., 2013), kDNA-PCR-RFLP (El Hamouchi et al., 2017), PCR-RFLP analysis of the *N*-acetylglucosamine-1-phosphate transferase (*nagt*) gene (El Hamouchi et al., 2018), and multilocus sequence typing (MLST) (El Mazini et al., 2023). However, the diversity of this species in Morocco has not yet been explored at the genomic level. That being said, the epidemiological profile of *L. infantum* is changing, with a remarkable expansion into previously non-endemic regions or into geographical areas where *L. tropica* and *L. major* are circulating (Mhaidi et al., 2018; Ait Kbaich et al., 2017). In addition, the increasing number of cases caused by this species documents a shift from sporadic to endemic transmission for the cutaneous form (El Mazini et al., 2022). These evolving clinical and geographical dynamics underscores the need for a more comprehensive and integrated approach to explore the population structure, the transmission dynamics and diversity of *L. infantum* strains.

Therefore, the objective of this study is to initiate for the very first time the exploration of the genomic diversity of *Leishmania infantum* in Morocco using a population originating from different vertebrate hosts, associated with different clinical forms, and collected from various geographical regions through comparative genomics.

## 2 Materials and methods

### 2.1 Ethical considerations

Consent was obtained from all participants included in this study: directly from adult participants (CL) and, for young children (VL), from their parents or legal guardians. The study involving human participants was approved by the Ethics Committee for Biomedical Research (CERB) of the Faculty of Medicine and Pharmacy, Mohammed V University (IORG 0006594; approved on 16 April 2019).

In addition to the procedures applied to human participants, canine samples are not subject to review by an animal ethics committee in Morocco. Nevertheless, all procedures involving dogs were conducted in accordance with the recommendations of the Institut Pasteur du Maroc, including: (i) the Charter of Fundamental Rights of the European Union (2000/C 364/01) and (ii) Council Directive 86/609/EEC of 24 November 1986 on the protection of animals used for experimental and other scientific purposes.

### 2.2 Sampling and culture of *Leishmania infantum*

The patients included in the sampling were children affected by visceral leishmaniasis (VL) who were sampled as part of the disease diagnostic process. Consent for participation and sample use was obtained from their parents or legal guardians prior to inclusion in the study. Bone marrow sampling was performed by iliac crest puncture by a pediatrician at the hospital. As for the patients presenting suspected cutaneous leishmaniasis (CL) lesions, who attended health centers in the northern part of the country (Taza, Ouazzane, and Taounate), were sampled as part of the diagnostic process. As for the canine samples, they were collected as part of surveys conducted in several regions of Morocco, under local anesthesia and aseptic conditions. The popliteal lymph node was sampled using a 5 mL syringe containing sterile physiological saline solution and antibiotics (penicillin and streptomycin).

Over thirty samples of *Leishmania* were collected for the study and isolated on NNN medium (Novy–MacNeal–Nicolle) and incubated at 25 °C. Established cultures were expanded in RPMI 1640 medium (Biowest, Nuaille, France), supplemented with 10–20% heat-inactivated foetal bovine serum (56 °C for 30 minutes, Eurobio, Les Ulis, France), antibiotics (100 IU/ml penicillin and 1 mg/ml streptomycin (Biowest, Nuaille, France) and 2 mM of the amino acid L-glutamine (Biowest, Nuaille, France).

### 2.3 DNA extraction, sequencing, quality control and sequence trimming

Nine strains were successfully isolated and total DNA was extracted using the DNeasy Blood & Tissue Kit (QIAGEN, Hilden Germany), following the manufacturer’s instructions. All strains were identified as *L. infantum* by PCR-ITS1-RFLP using the HaeIII enzyme (Schönian et al., 2003). DNA quality was assessed by determining the DNA Integrity Number (DIN) through the analysis of 20 ng of DNA on a TapeStation 4200 (Agilent). One microgram of genomic DNA was used to prepare a library for whole-genome sequencing on the automated platform Bravo NGS Workstation (Agilent), using the Illumina PCR-free “TruSeq DNA” library preparation kit, according to the manufacturer’s instructions. After normalization and quality control, the libraries were sequenced on an Illumina HiSeqX5 platform (Illumina Inc., CA, USA) at the National Center for Human Genetics Research (CEA, Evry, France), generating 150 bp paired-end reads. The sequence quality parameters were evaluated throughout the sequencing run. Standard bioinformatics analysis of the sequencing data was performed using the Illumina pipeline to generate a FASTQ file for each sample.

We successfully sequenced five strains of *L. infantum*; one strain of VL, two strains of CL (one of which was isolated from an immunocompromised patient, CL/HIV), and two strains of canine leishmaniasis. Additionally, we retrieved a VL genome sequence (ERR524883) isolated in Morocco, sequenced using an Illumina platform at the Wellcome Sanger Institute, reported by Franssen et al., 2020, from the ENA database (https://www.ebi.ac.uk/ena/browser/search). We included it as one of the samples as it is the only Moroccan VL strain publicly available to extend our study so that we can have two samples of each disease form (**Table 1**).

**Table 1:** Characteristics of the six strains studied, including the disease form, age, sex, year of isolation, geographic origin, immune status and number of passages.

Sample	SLH16	ERR524883	FA23	LC_86	LEM00	LU22
Disease form	VL	VL	CL	CL	CanL	CanL
Age	9	–	35	45	3	4
Sex	Female	–	Female	male	male	male
Year of isolation	2016	1967	2023	2022	2000	2022
Geographic origine	North	North	North (Taounate)	North	North	North-west (Rabat)
Immune status	Immunocomp-etent	–	Immunocom-petent	HIV-coinfcetd	–	–
Number of passages	4 passages, after thawing	–	5 passages	5 passages	4 passages, after thawing	5 passages

After receiving the sequenced data, the quality of the sequences was assessed and reads were trimmed using the fastp tool (Chen et al., 2018) and the FastQC program (v0.11.7).

### 2.4 Sequence analysis

All results and figures presented in this paper were generated using the Genome Instability Pipeline (GIP) and giptools version 1.0.9 (Späth and Bussotti, 2022).

All GIP outputs were used as input for giptools, a suite of modules that allows comparison of samples processed by GIP, visualization of changes in chromosome copy number, gene copy number, and the frequency of small nucleotide polymorphisms (SNPs). The GIP code is maintained and freely distributed on GitHub: https://github.com/giovannibussotti/GIP. The full documentation for GIP and giptools, including a description of all options, is available at: https://gip.readthedocs.io/en/latest/.

### 2.5 Read alignment

Whole-genome sequencing (WGS) reads were mapped by GIP using BWA-mem (version 0.7.17) (Li and Durbin, 2009) with the -M option to label shorter split hits as secondary, using the reference genome *Leishmania infantum* GCA_900500625 (Howe et al., 2020). The alignment files were then sorted, indexed, and reformatted by GIP using Samtools (version 1.8) (Li and Durbin, 2009). Finally, duplicate reads were removed by GIP using Picard MarkDuplicates (http://broadinstitute.github.io/picard) (version 2.18.9) with the option ‘VALIDATION_STRINGENCY = LENIENT’.

### 2.6 Sequencing coverage ratio

To ensure accurate comparisons, we normalized the coverage values for each genomic segment. The normalization process involved calculating the ratio of the actual coverage for a given segment to the median coverage across all segments. These steps were performed using BEDTools (version 2.31.0) in conjunction with R (tidyverse package). The coverage ratio between isolates was calculated using the ‘binCNV’ module of giptools. Gene coverage scores were normalized by the median chromosomal coverage to highlight amplifications or depletions relative to the chromosome copy number. Consequently, CNV detection was based on normalized coverage ratios rather than absolute read counts, enabling reliable comparisons among isolates despite variation in sequencing depth. The coverage ratio between isolates was calculated using the ‘geCNV’ module of giptools.

### 2.7 Single nucleotide polymorphism

Single Nucleotide Polymorphisms (SNPs) were identified using Freebayes (version 1.3.2) (Garrison and Marth, 2012). To ensure robust variant detection across samples with different sequencing depths, SNPs were subjected to quality filtering within the GIP pipeline using criteria based on allele frequency, supporting read counts, and mapping quality, retaining only high-confidence variants for downstream analyses. The impact of SNPs on coding sequences was predicted using SnpEff (version 4.3t) (Cingolani et al., 2012). To assess the number of unique and shared SNPs between all isolates, we generated an UpSet plot using the ‘UpSetR’ package.

### 2.8 Taxonomic screening of reads with Kraken2 (hybrid pre-assessment)

For each isolate (ERR524883, LEM00, FA23, LC86, LU22, SLH16), we used the raw paired-end FASTQ files (R1/R2). Taxonomic assignments were obtained with Kraken2 using the PlusPF-8 prebuilt database_20250714.tar.gz; release 2025-07-14), which includes Bacteria, Archaea, Viruses, Protozoa (thereby covering Leishmania spp.), Fungi and Human. For each sample we summed the percentages assigned to species whose scientific names begin with “*Leishmania *…”, we then calculated (i) within-*Leishmania* composition by normalizing each *Leishmania* species’ percentage to the total *Leishmania* percentage in that sample, and (ii) the fraction of *L. donovani* within *Leishmania* = (*L. donovani* % of all reads)/(Total *Leishmania* % of all reads) × 100.

## 3 Results

### 3.1 Alignment statistics of the strains

The alignment statistics of the six *L. infantum* strains against the reference genome (Leishmania_infantum_gca_900500625.LINF.48) are presented in **Table 2**. The proportion of aligned reads ranges from 81% for ERR524883 to 96% for SLH16, FA23, and LU22. The sequencing depth ranged from 33x to 70x across samples. The high alignment rates and deep coverage (>50x) in most sequenced strains support robust detection of genomic variation, including somy estimation and SNP calls. Even the lowest depth (33x) in ERR524883 remains above the typical threshold for reliable whole-genome variant analysis in *Leishmania*.

**Table 2:** Alignment statistics of all samples.

Samples	Total reads	Aligned reads passing the illumina filter (PFI)	% aligned reads (PFI)	Sequencing depth
SLH16	16757035	16163688	96	49
ERR524883	10263133	8321832	81	33
FA23	16133260	15486480	96	66
LC_86	17797742	16336406	92	70
LEM00	18438526	17479805	94	55
LU22	11959778	11505388	96	50

To evaluate the genomic diversity of the *L. infantum* strains studied, we conducted several analyses in the following order: chromosomal copy number variation, segment-level copy number variation (binCNV), gene-level copy number variation (geCNV), single nucleotide polymorphisms (SNPs) analysis, principal component analysis (PCA), and finally the genomic distance. This approach was designed to maximize our understanding of the genomic diversity of this species.

### 3.2 Karyotypic profile of the strains

**Figure 1:**
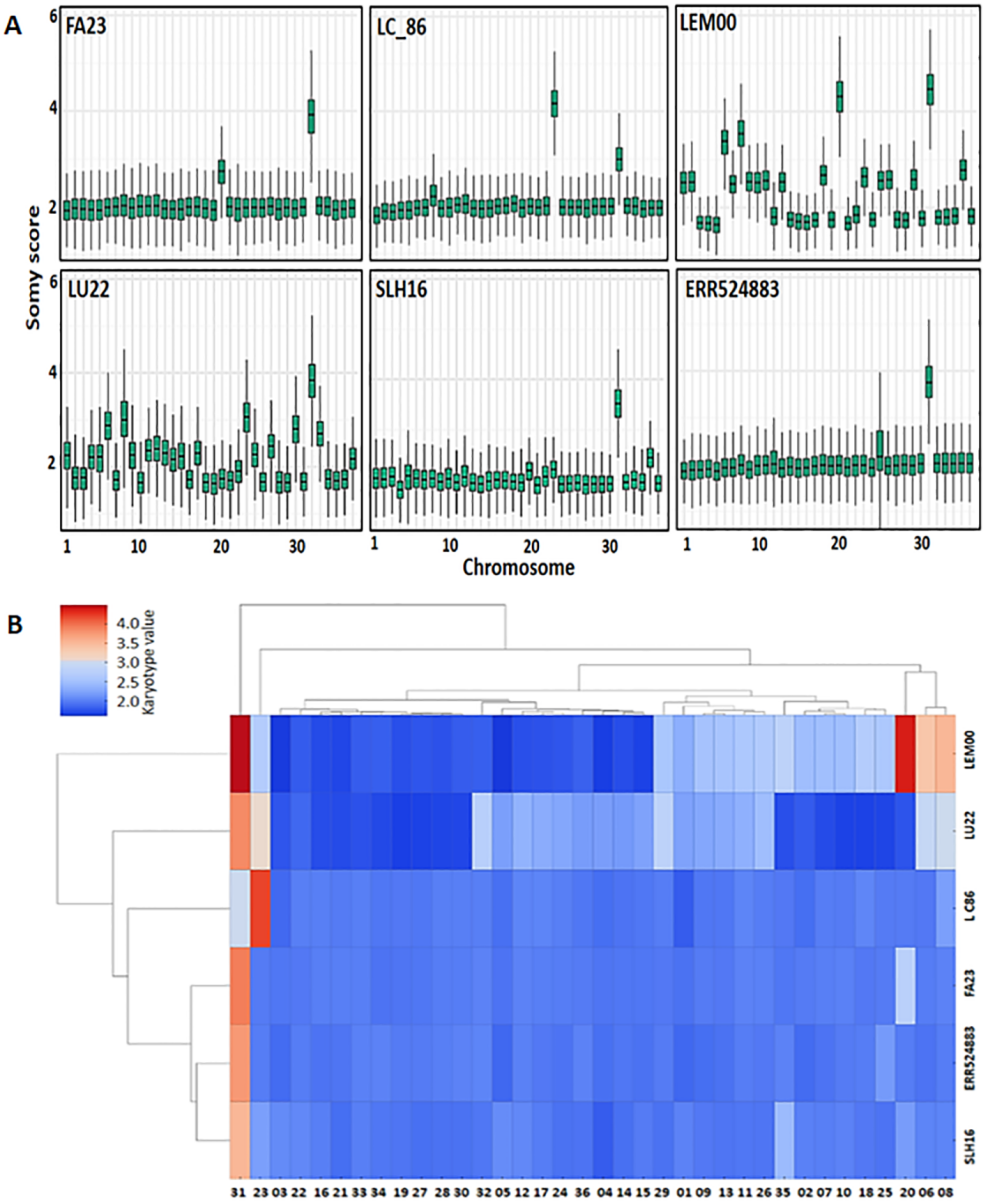
**(A)** Aneuploidy of the six strains: Box plots representing the distributions of somy scores for each chromosome in the indicated samples. **(B)** Heatmap of estimated chromosomal somy scores across six *L. infantum* strains. Each row represents a strain (LEM00, LU22, LC_86, FA23, ERR524883, SLH16), and each column corresponds to one of the 36 chromosomes. Colors indicate the estimated somy score for each chromosome in each strain, with blue shades representing disomy (≈2), and warmer colors (yellow to red) indicating higher copy numbers (trisomy to tetrasomy and beyond). The dendrogram at the top clusters strains based on their karyotypic profiles, while the left-side clustering groups chromosomes with similar copy number patterns across strains.

Chromosomal somy analysis revealed variable patterns of aneuploidy across the six *L. infantum* strains (**Figure 1**). As expected, the majority of chromosomes in most strains displayed disomy (somy score ≈2), consistent with the canonical *Leishmania* karyotype. Notably, chromosome 31 exhibited tetrasomy (somy score ≈ 4) across all strains, reaffirming its well-documented amplification in *Leishmania* species, potentially due to its role in parasite fitness and virulence.

The two canine strains, LEM00 and LU22, demonstrated the highest levels of karyotypic variability. LEM00 exhibited extensive aneuploidy, with multiple chromosomes showing trisomy or tetrasomy, and chromosome 20 approaching pentasomy. LU22 showed similar karyotypic instability, particularly for chromosomes 6, 8, 23, and 27. This pronounced chromosomal plasticity in canine strains may reflect adaptation to the canine host, environmental pressures within the zoonotic reservoir, or be a result of culture adaptation as previously described (Prieto Barja et al., 2017).

In contrast, the visceral strains SLH16 and ERR524883 exhibited relatively stable chromosomal profiles, with disomy predominating aside from the consistent tetrasomy of chromosome 31. The cutaneous strain FA23 displayed a mostly stable karyotype, whereas LC_86 (isolated from an HIV-positive patient) showed moderate somy variability on chromosomes 23 and 31. These findings collectively suggest that host type and immune status may influence the extent of chromosomal copy number variation, with canine strains showing greater genomic plasticity compared to those isolated from human visceral cases.

We next analysed the six samples using hierarchical clustering based on their somy profiles (**Figure 1B**). The dendrogram was obtained using the “overview” model from giptools via Euclidean distances based on the aneuploidy profile. The results revealed two major groups among the six *L. infantum* strains. The first cluster included SLH16, ERR524883, and FA23, which displayed overall chromosomal stability with predominantly disomic chromosomes and consistent tetrasomy of chromosome 31. This grouping reflects conserved karyotypic features, particularly among the two visceral strains (SLH16 and ERR524883), and suggests limited chromosomal plasticity in these isolates. The second cluster grouped LC_86 with the two canine strains, LU22 and LEM00, which showed markedly higher levels of aneuploidy across multiple chromosomes, including chromosomes 8, 20, 23, and 32. This distinct karyotypic profile suggests that chromosomal copy number variation may be associated with host type (canine or immunocompromised human) and may reflect adaptive genomic responses to host or environmental pressures, including culture adaptation.

### 3.3 Gene level CNV profiling

**Figure 2:**
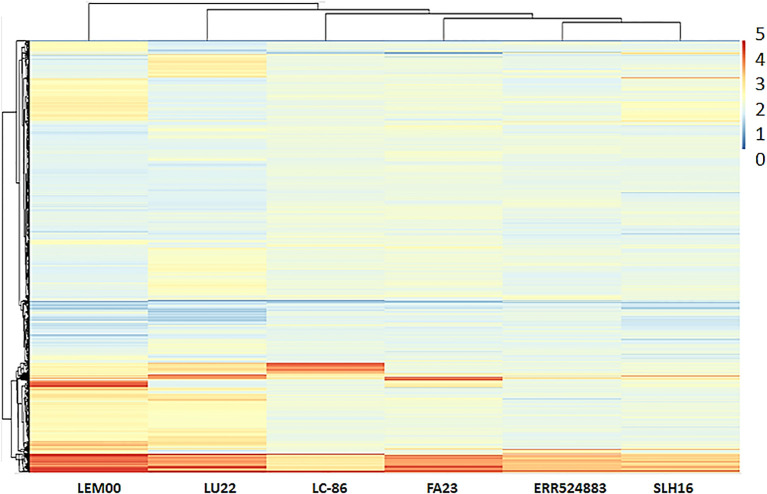
Heatmap of gene-level copy number variation (geCNV) across six *Leishmania infantum* strains. Columns represent individual strains, while rows correspond to genes ordered by hierarchical clustering. The color scale reflects normalized copy number values per gene, calculated as the gene’s average coverage divided by the median chromosomal coverage. Warmer colors (red) indicate gene amplifications, and cooler colors (blue) indicate depletions. The dendrogram at the top groups strains based on their geCNV profiles.

We next analyzed gene-level copy number variation (geCNV) across the six *L. infantum* strains using the ‘overview’ module of giptools, which computes normalized gene copy number values across the genome. Gene copy number was derived by normalizing the average coverage of each gene against the median coverage of its corresponding chromosome, as reported by GIP. The resulting normalized values were visualized as a heatmap (**Figure 2**), with samples clustered according to the similarity of their gene CNV profiles.

The dendrogram revealed two main clusters, consistent with the above somy and gene CNV analyses. SLH16 and ERR524883 (both visceral strains), along with the cutaneous strain FA23, formed a distinct group with relatively stable gene copy number patterns. In contrast, the two canine strains, LU22 and LEM00, appeared the most divergent, displaying numerous gene amplifications (in red) and depletions (in blue). The cutaneous/HIV strain LC_86 also exhibited a distinct pattern, though to a lesser extent than the canine strains.

This clustering reinforces the patterns observed in chromosomal somy and gene CNVs, suggesting that gene-level dosage alterations may be linked to broader karyotypic changes. Moreover, the extensive gene CNVs observed in LEM00 and LU22 may reflect host-specific adaptation to the canine reservoir via genomic plasticity.

In our collection of strains, we have representatives of three forms of the disease: canine leishmaniasis represented by LEM00 and LU22, cutaneous leishmaniasis represented by FA23 and LC_86, and visceral leishmaniasis represented by SLH16 and ERR524883. The diversity of isolate sources constitutes an important aspect of this study, allowing us to explore genomic variation associated with different clinical manifestations of *Leishmania infantum* in Morocco.

We assessed gene dosage changes against the reference genome and between these pairs of strains using the ‘geCNV’ module of giptools to examine genes with CNVs corresponding to amplifications or deletions in each form of the disease.

The observed series of CNVs represent amplification/depletion of entire genes:

Canine leishmaniasis (LU22, LEM00): Among the amplified genes in the canine strains, the majority are coding for hypothetical proteins, followed by genes encoding amastins (LINF_080011900 and LINF_080012000) with a normalized coverage ratio of 3.0 and 3.8 respectively, which show the highest amplification values. Finally, amplification was also noted for genes encoding phosphoglycan β1–3 galactosyltransferase, ATG8, gp63, Hsp70, and histones (full list is available in **Supplementary Table 1**).

Visceral leishmaniasis (SLH16 and ERR524883): In the visceral leishmaniasis isolates, the highest levels of amplification were observed for two copies of the proteophosphoglycan PPG3 gene (LINF_350010200 and LINF_350010100), with normalized coverage ratios of 26.1 and 9.1, respectively, relative to the reference genome. Additional amplified genes included a putative amastin surface glycoprotein (LINF_310009800), with a normalized coverage ratio of 5.4, and two phosphoglycan β1,3-galactosyltransferase genes (LINF_020006900 and LINF_020007000), with ratios of 3.2 and 3.6, respectively. Amplification was also detected in genes encoding elongation factor 2 (LINF_360006900, LINF_360007100, and LINF_360006800), α-tubulin, and a surface antigen (LINF_040007000) (the complete list is provided in **Supplementary Table 1**).

Cutaneous leishmaniasis (FA23 and LC_86): Among the amplified genes detected in the cutaneous isolates, the most prominent signal was observed for a gene encoding a surface antigen (LINF_120014600), with a normalized coverage ratio of 3.2. This was followed by a putative amastin gene (LINF_340034400) with a ratio of 2.7. Additional amplified genes included ATG8 (LINF_090007100) and phosphoglycan β1 (LINF_350005000) (the complete list is provided in **Supplementary Table 1**).

Read depth analysis across the different strains further revealed notable gene depletions. In canine strains, five copies of hypothetical proteins located on different chromosomes were depleted, along with four copies of the calpain-like cysteine peptidase genes (LINF_270010300, LINF_270010400, LINF_270010500, and LINF_270010600) on chromosome 27. In strains associated with visceral leishmaniasis (VL), four copies of ATG8-coding genes on chromosome 19 (LINF_190013300, LINF_190013500, LINF_190014000, and LINF_190014100), and two copies of genes encoding the putative RNA-binding protein UPB1 on chromosome 25 (LINF_250010100 and LINF_250010200) were found to be depleted. Four hypothetical proteins located on various chromosomes were also affected. In cutaneous leishmaniasis (CL) causing strains, three copies of the calpain-like cysteine peptidase genes (LINF_270010400, LINF_270010500, and LINF_270010600) were depleted on chromosome 27, while amastin surface glycoprotein gene depletions were observed in two copies on chromosome 8 (LINF_080012100 and LINF_080012200) and one copy each on chromosome 31 (LINF_310009800) and chromosome 34 (LINF_340024000). Furthermore, three ATG8 gene copies (LINF_190013400, LINF_190014000, and LINF_190014200) were depleted on chromosome 19 (Additional details in **Supplementary Table 2**).

Regarding the year of isolation, our samples were collected over different years. However, across all analyses performed, we did not observe any clustering or grouping of isolates according to their year of isolation. This indicates that temporal variation was not a major factor driving the genomic patterns observed in our dataset.

### 3.4 Single nucleotide polymorphism analysis

**Figure 3:**
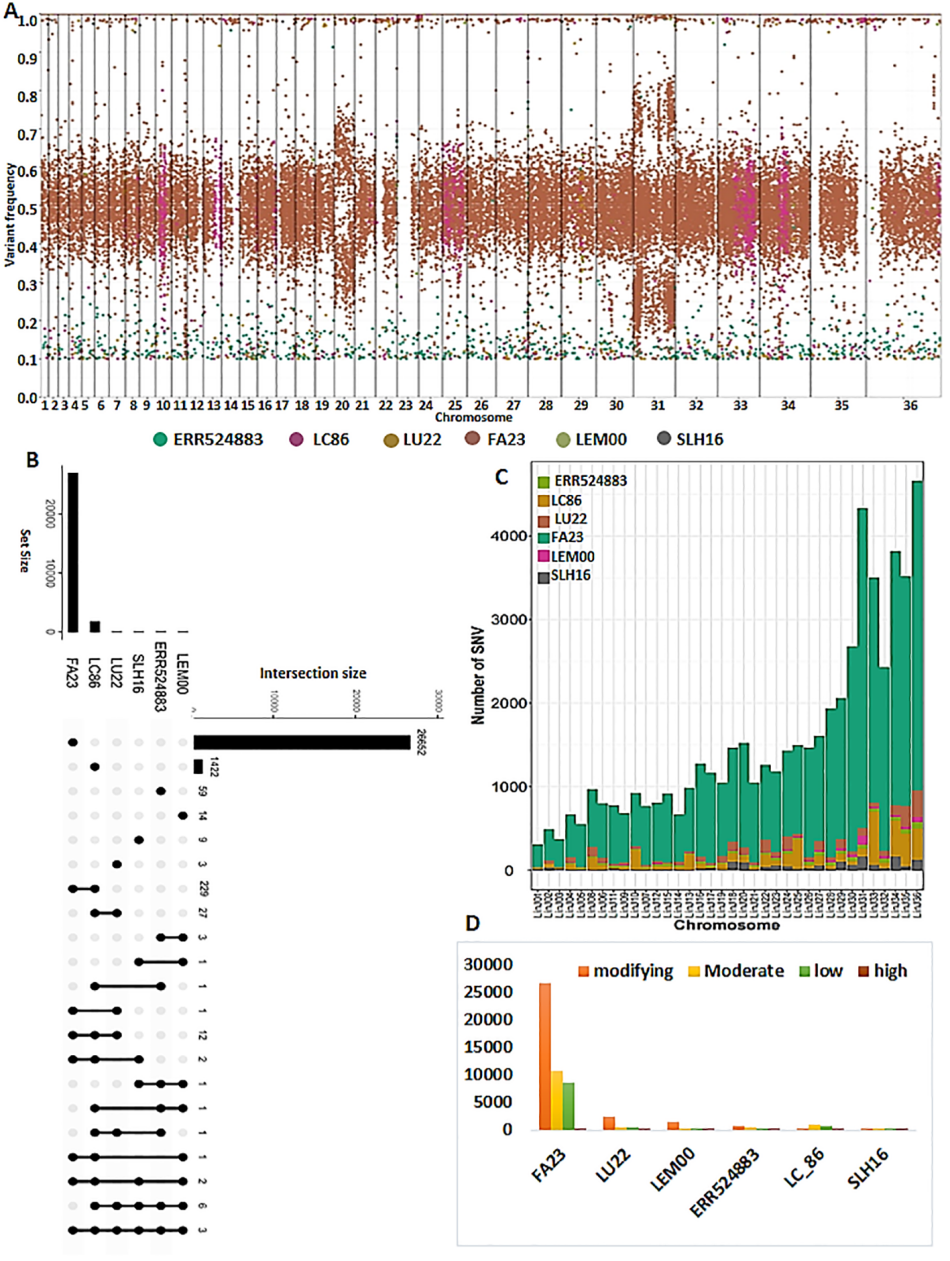
**(A)** Genome-wide distribution of SNP allele frequencies across six *Leishmania infantum* strains. Each point represents a single SNP, plotted by its allele frequency (y-axis) and chromosomal location (x-axis). SNPs were called using Freebayes relative to the GCA_900500625 reference genome. Colors distinguish strains: LU22 (orange), FA23 (brown), SLH16 (dark grey), LEM00 (light green), LC_86 (pink), and ERR524883 (green). **(B)** UpSet plot illustrating shared and unique SNPs across all 6 isolates. **(C)** Histogram of SNP number of the six samples. The x-axis represents the 36 chromosomes and the y-axis the number of SNP. **(D)** Distribution of SNPs by predicted genomic impact across the six *Leishmania infantum* strains: modifying, low, moderate, and high.

We continued our genome analysis by investigating single-nucleotide polymorphisms (SNPs) in each of the *L. infantum* strains relative to the reference genome. Variant allele frequencies were analysed genome-wide to assess strain-specific polymorphism patterns across all 36 chromosomes (**Figure 3A**).

**Figure 3A** illustrates the distribution of variant frequencies across the analyzed genomes. The FA23 strain (brown) exhibits a markedly higher density of SNPs across nearly all chromosomes, with particularly elevated variation on chromosomes 20 and 31. This pattern includes numerous mid-frequency variants (∼0.5), which may indicate high heterozygosity or potential hybrid origin. The LC_86 strain (pink) also shows elevated SNP density relative to the other isolates, though less pronounced than FA23. In contrast, the remaining strains SLH16, ERR524883, LU22, and LEM00 exhibit lower SNP counts, consistent with greater similarity to the reference genome or reduced genetic variability. The uneven chromosomal distribution of SNPs suggests localized hotspots of mutation or recombination, while the concentration of FA23’s variants at intermediate frequencies points to genomic mosaicism that warrants further investigation.

We used the UpSet plot to assess the number of unique and shared SNPs among all isolates; the UpSet plot shows the shared SNPs for a given combination of the isolates. The most diverse sample FA23 had the highest number of unique SNPs (26652), followed by LC86 the co-infected cutaneous-HIV isolate (1422). Only 229 SNPs are shared between these two samples (**Figure 3B**).

The total number of SNPs in the six strains compared to the reference genome is 49550, with distribution across the samples ranging from 45759 in FA23 to 604 in LEM00. The majority of SNPs are found in intergenic regions, ranging from 319 (LEM00) to 26558 (FA23) (**Table 3**).

**Table 3:** Total number of SNPs per strain and their distribution across intergenic and genic regions.

SNPs	FA23	LC_86	LU22	SLH16	ERR524883	LEM00
Total number of SNPs	45759	4294	2395	1469	988	604
Intergenic SNPs	26558	2526	1403	817	411	319
Genic SNPs	19201	1768	992	652	577	285
Genes with SNPs	5679	912	640	476	523	251
Synonymous variants	8471	803	416	276	132	100
Non-synonymous variants	10673	958	572	375	438	184
stop-gained variants	33	3	2	–	6	–
stop_lost & splice_region_variant	12	3	2	1	–	1
splice_region_variant & stop_retained_variant	9	1	–	–	–	–
start_lost	3	–	–	–	1	–

Within genic regions, SNPs are further categorized into synonymous, non-synonymous, and stop-gained variants.

Regarding genic SNPs, non-synonymous mutations were more frequent than synonymous mutations across all strains, with counts ranging from 184 in LEM00 to 10,673 in FA23. The non-synonymous SNPs identified in our dataset were predominantly missense variants, which involve single or multiple base changes that alter the encoded amino acid sequence without affecting protein length.

In addition, 41 stop-gained variants were detected. These variants result from nucleotide substitutions that introduce a premature stop codon, leading to truncated transcripts. Seventeen variants were classified as stop-lost and splice-region variants; stop-lost variants arise from changes in at least one nucleotide of the termination codon, resulting in an elongated transcript, while splice-region variants correspond to nucleotide changes occurring within splice-site regions (1–3 bp of the exon or 3–8 bp of the intron).

Ten variants were annotated as splice-region variants with stop-retained variants, in which nucleotide changes occur within splice-site regions while the termination codon is altered but remains functional. Finally, four start-lost variants were identified, caused by nucleotide substitutions affecting the canonical start codon, potentially impairing translation initiation (**Table 3**; **Supplementary Table 3**).

The histogram shows the number of SNPs of each isolate per chromosome. The sample FA23 showed the highest number of SNPs in all chromosomes. The sample LC_86, second to FA23 showed high density across most of the chromosomes, indicating the divergence of these two isolates from the rest. Meanwhile, the other isolates showed fewer SNPs confirming previous findings (**Figure 3C**).

We then classified the identified SNPs by impact, distinguishing high-impact variations from low-impact variations. High-impact variations refer to changes likely to have significant consequences on protein structure or function, while low-impact variations represent changes less likely to cause major disruptions, with more moderate effects on protein function.

**Figure 3D** shows that, in general, for all strains, variations with a modifier impact (primarily located in intergenic regions) are more numerous than variations with a moderate impact, while low and high impact variants are the least frequent in all strains (**Figure 3D**).

**Figure 4:**
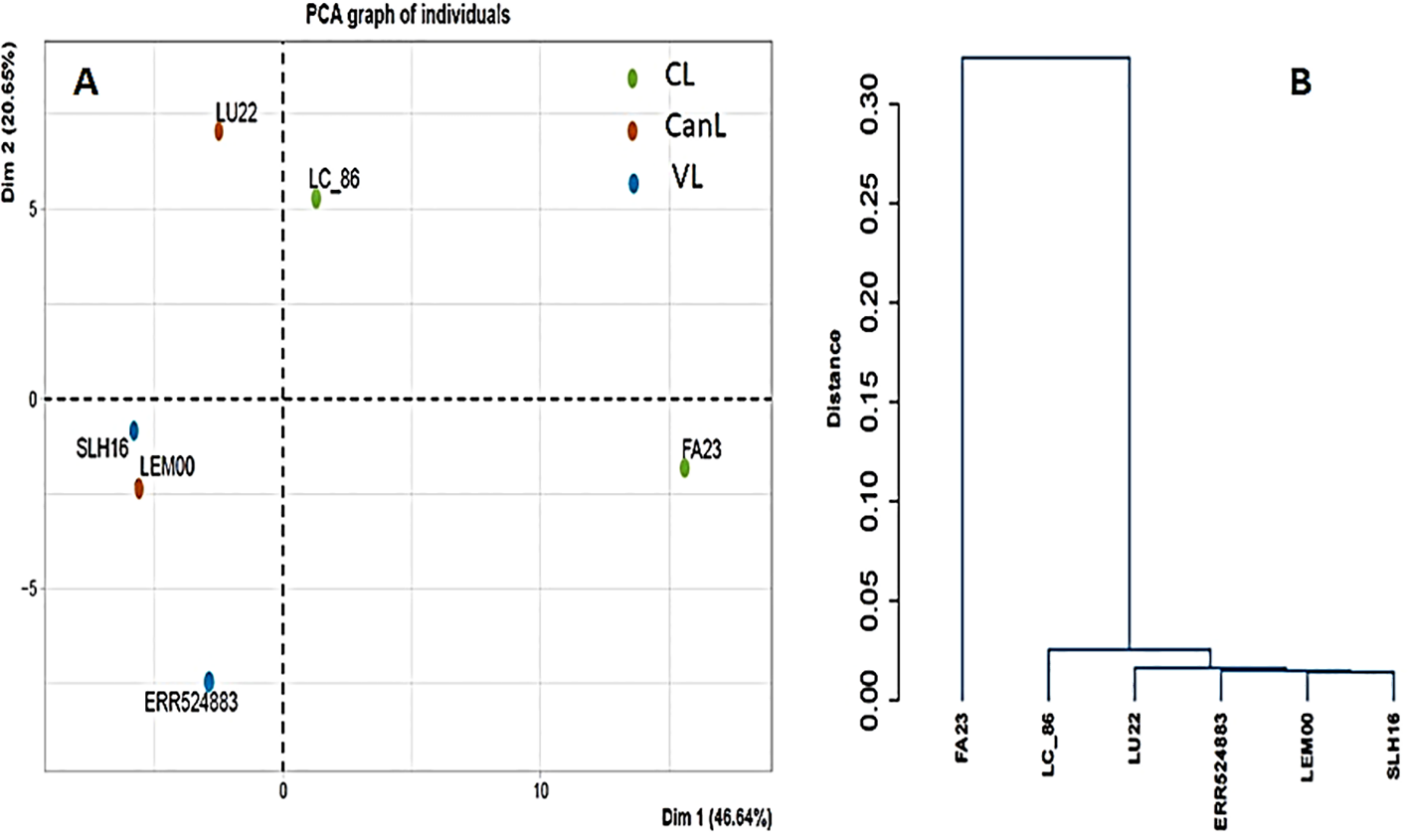
Genomic distance-based clustering of six *Leishmania infantum* strains using SNP data. **(A)** Principal component analysis (PCA) plot showing genetic divergence among strains along the first two principal components (Dim 20.65% and Dim 46.64%). **(B)** Hierarchical clustering dendrogram based on pairwise genomic distances.

To assess the overall genomic relatedness among the six *L. infantum* strains, we used the genomeDistance module of giptools, which computes pairwise distances based on SNP data. These distances were then used to generate both a principal component analysis (PCA) plot and a hierarchical clustering dendrogram (**Figure 4**).

Both the dendrogram and the PCA graph show the genomic distance between the cutaneous strains FA23 and LC_86, which form two distinct branches. The group of four strains consists of two subgroups: one subgroup contains three strains, two from visceral leishmaniasis (SLH16, ERR524883) and one canine strain (LEM00), while the other subgroup consists of LU22 (**Figure 4**).

These two analyses indicate that the two cutaneous strains are distantly related to the other strains of visceral and canine origin. This highlights the importance of further exploration of these dermotropic strains.

### 3.5 LC_86; the co-infected CL isolate

This strain isolated from a cutaneous leishmaniasis patient with HIV co-infection exhibited significant diversity, as demonstrated by multilocus sequence typing (MLST) (El Mazini et al., 2023). This diversity was further confirmed through genomic analyses, reinforcing previous observations on *L. infantum* strains in HIV co-infected individuals. Notably, zymodeme characterization in such patients has revealed greater heterogeneity compared to strains from immunocompetent leishmaniasis cases or natural canine reservoirs (Gramiccia et al., 1995; Cruz et al., 2002; Chicharro et al., 2003; Cortes et al., 2014). These findings suggest that HIV co-infection may drive unique parasite population dynamics, possibly due to altered host immune pressures.

### 3.6 FA23 is a highly divergent strain

**Figure 5:**
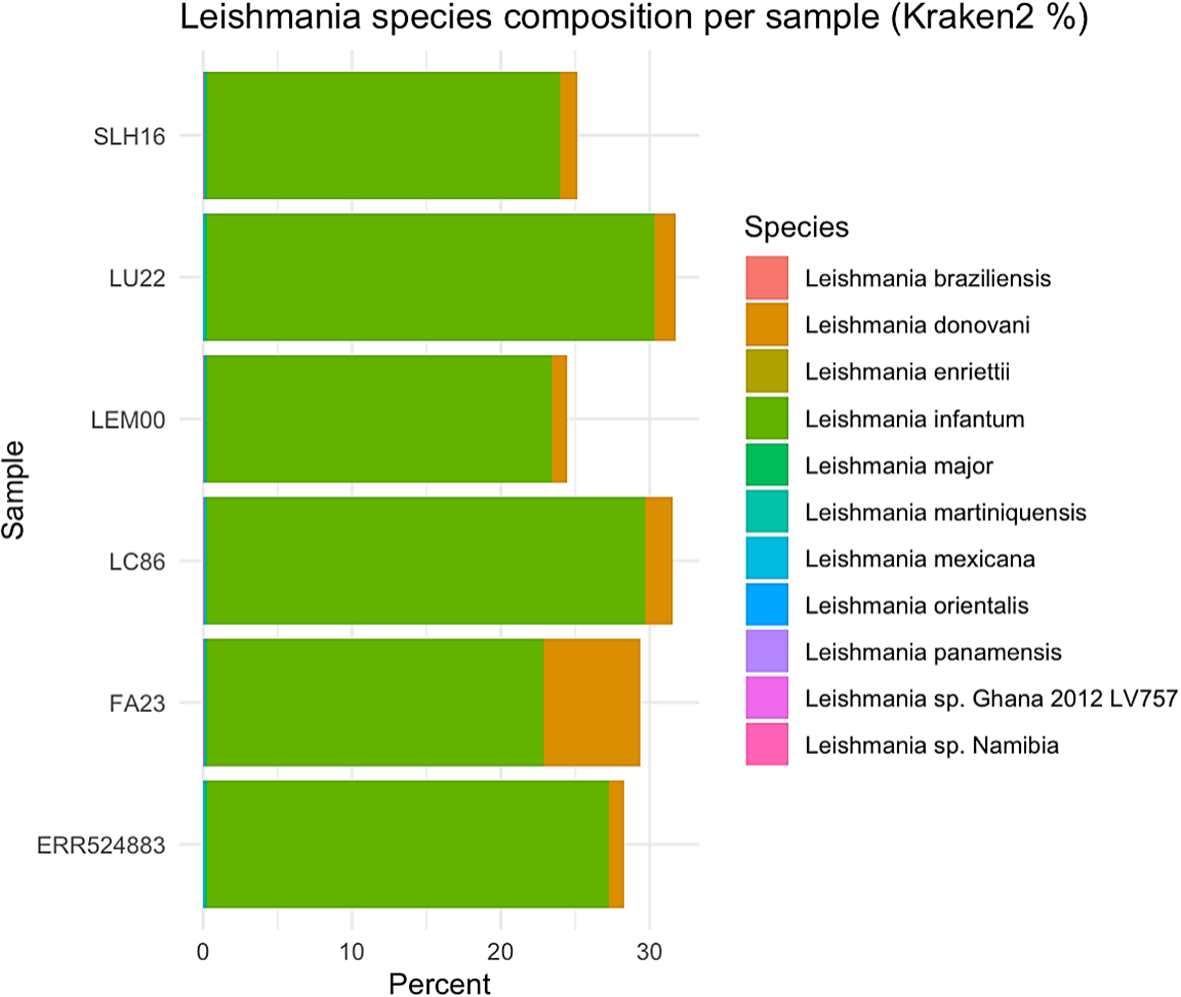
*Leishmania* species composition per sample (Kraken2%), normalized within the genus. For each sample, the proportions of reads assigned to *Leishmania* species are scaled to sum to 100%, highlighting differences in the relative contribution of *L. donovani* across isolates. The *L. infantum* Illumina control (ERR1913337; PRJEB20254) is included as a positive control.

To explore the possibility of mixed ancestry in our isolates, we performed taxonomic classification of Illumina reads using Kraken2 against a protozoa-inclusive database (PlusPF-8). Within the *Leishmania* genus, all isolates were predominantly assigned to *L. infantum* (**Figure 5**). As a positive control, we included Illumina reads from a publicly available *L. infantum* dataset (ERR1913337, deposited under PRJEB20254), processed through the same Kraken2 workflow. This control showed near-exclusive assignment to *L. infantum* within the genus, supporting the specificity of the classification under our database and parameters and providing a baseline expectation for a non-hybrid *L. infantum* sample.

For clarity, **Figure 5** shows the relative species composition within *Leishmania* (i.e., *Leishmania* assignments scaled to 100% per sample). Under this representation, isolate FA23 displayed a markedly higher contribution of *L. donovani* (22.21%) compared with the other isolates (3.74–5.86%), while *L. infantum* accounted for the remaining fraction (77.79% in FA23 vs. ~94–96% in the other isolates). Minor assignments to other *Leishmania* species were negligible across samples, the corresponding values are provided in **Supplementary Table 4**. Overall, this taxonomic profile highlights FA23 as a clear outlier due to its elevated *L. donovani* signal. Although Kraken2 classification alone cannot distinguish true mixed ancestry from cross-assignment between closely related taxa or low-level contamination, the consistent contrast with the ERR1913337 control supports that the FA23 pattern is not an artifact expected for a typical *L. infantum* dataset and is compatible with a hybrid-related signal.

## 4 Discussion

*Leishmania infantum* is a clinically important *Leishmania* species endemic in various areas around the world, both from veterinary and public health perspectives. In Morocco, it is responsible for canine and human leishmaniasis manifesting as both visceral and cutaneous forms of the disease. Despite its clinical significance in the country, no whole-genome sequencing studies have previously been conducted on local isolates of this species. Therefore, we undertook, for the first time, an exploration of the intraspecific genomic diversity of *L. infantum* in Morocco.

Whole-genome sequencing offers high-resolution insights into *Leishmania* strains, enabling detailed analysis of karyotype variation (aneuploidy), gene copy number variation (CNV), and single nucleotide polymorphisms (SNPs) (Späth and Bussotti, 2022). Genomic exploration reveals the complexity of the *Leishmania* genome and enhances our understanding of gene function (Downing et al., 2011; Carnielli et al., 2018).

Our results revealed aneuploidy in the genomes of the six *L. infantum* strains, consistent with previous reports (Rogers et al., 2014; Teixeira et al., 2017; Dumetz et al., 2017; Bussotti et al., 2018; Patino et al., 2021; Bruno et al., 2024). Chromosome amplifications may enhance the parasite’s fitness and facilitate host adaptation, although karyotypic changes emerging during culture adaptation cannot be ruled out. This phenomenon has been widely observed across Leishmania species (Dumetz et al., 2017; Prieto Barja et al., 2017; Cuypers et al., 2018; Bussotti et al., 2018; Patino et al., 2019).

In our study, the canine strains exhibited the highest karyotypic instability, with aneuploidy affecting multiple chromosomes, likely reflecting adaptive responses to the canine host environment. Chromosome amplification patterns are known to be non-random, species-dependent, and strain-specific, pointing to selection driven by parasite-intrinsic genetic factors (Imamura et al., 2016; Dumetz et al., 2017; Bussotti et al., 2018).

Finally, since all isolates were cultured before sequencing, culture adaptation may have influenced their aneuploidy profiles, which are known to fluctuate rapidly in response to environmental changes and stress (Sterkers et al., 2011; Dumetz et al., 2017; Bussotti et al., 2018; Lachaud et al., 2014).

In contrast to fast karyotypic changes observed in culture, gene copy number variations (CNVs) are selected only during long-term culture (> 20 passages). Therefore, gene copy number variations are much less dynamic and may reveal modifications in gene dosage that have been selected in the field (Bruno et al., 2024).

In our study, the genes with the highest amplifications across all strains are encoding for important virulence factors, including various amastin family members or genes involved in the synthesis of phosphoglycan virulence factors known to enhance infectivity, indicating their likely importance in host-specific adapting to (Urrea et al., 2018; Bruno et al., 2024). These genes exhibited the strongest amplifications in the visceral strains, highlighting the severity of this form and illustrating the role of CNVs as possible biomarkers to elucidate strain-specific pathogenicity. As shown previously by Rogers et al., 2011; Dumetz et al., 2017; Prieto Barja et al., 2017; and Iantorno et al., 2017 the increase in gene copy number can lead to changes in gene expression in response to the host’s environmental conditions, thereby providing a potential genetic basis to explain the tropism of the disease.

Certain gene amplifications were found to be common across different clinical forms of leishmaniasis, possibly highlighting shared mechanisms of pathogenicity. For example, the gp63 gene, which encodes a key surface protease, was amplified in both canine and cutaneous strains. gp63 plays a crucial role as a virulence factor in *Leishmania*, by promoting rapid migration, facilitating internalization into host macrophages, and enhancing parasite survival within these immune cells (Olivier et al., 2012). In addition to gp63, other genes involved in stress response and *Leishmania* infectivity also showed amplification. The gene encoding surface antigen protein 2, known for its role in host–parasite interactions and immune evasion, was amplified in both cutaneous and visceral strains (Urrea et al., 2018). Similarly, the autophagy-related gene ATG8, involved in parasite survival under stress conditions and contributing to infectivity, was amplified in both canine and visceral strains (Giri and Shaha, 2019).

These shared gene amplifications suggest that despite the different clinical manifestations, *Leishmania* strains employ common molecular strategies such as enhancing virulence factors and adaptive stress responses to survive and thrive within their hosts. Understanding these shared genomic features could provide new targets for therapeutic interventions across multiple forms of the disease. Other gene amplifications appeared to be specific to particular disease forms, potentially indicating unique adaptations within distinct host environments. For instance, the hsp70 gene was found to be amplified exclusively in the canine strains. This amplification of hsp70 suggests a possible role in adaptation to the unique conditions within the canine host, potentially enhancing the parasite’s resilience and infectivity in this reservoir. Previous studies have identified hsp70 as a key hotspot in the environment-genotype interaction in field isolates of *L. donovani*, where it contributes significantly to the parasite survival and stress tolerance (Bifeld and Clos, 2015; Drini et al., 2016).

However, these strain and disease specific genomic patterns remain preliminary. Given the limited sample size in this study, further research with larger datasets is needed to validate these findings and to fully understand how such genetic variations influence disease progression, host specificity, and parasite fitness in different clinical contexts.

Single nucleotide polymorphisms (SNPs) are important genomic variations that may contribute to the adaptation of *Leishmania*. Our results identified diverse SNPs across all samples, with the majority located in intergenic regions. According to Cingolani et al. (2012), SNPs in these regions tend to have a modifier impact, even though their exact functional consequences are often difficult to predict or characterize. Nevertheless, such SNPs may influence critical processes such as gene regulation and translation, potentially facilitating adaptive response to environmental changes.

Synonymous SNPs have long been considered functionally insignificant since they preserve the primary sequence of proteins. However, evidence suggests that these variants may influence mRNA structure by affecting its stability, and eventually protein structure, and folding (Hunt et al., 2009). Such effects could contribute to the complexity of infectious diseases by modulating parasite virulence or host-pathogen interactions. Consequently, synonymous SNPs represent potential genetic markers for elucidating mechanisms of immune evasion and pathogenesis, underscoring their relevance in disease progression and host immune responses (Hunt et al., 2009).

Transport proteins, particularly mitochondrial ABC transporters and members of the ABC1 family, have been implicated in drug resistance mechanisms (Ouellette et al., 1998). In the cutaneous *Leishmania* strain isolated from the HIV positive patient who showed no clinical improvement after two doses of amphotericin B, two synonymous SNPs were identified in genes encoding these transporters. Although synonymous SNPs do not alter protein sequences, they may still influence treatment outcomes by affecting translational efficiency, protein folding, or mRNA stability (Laffitte et al., 2016). These findings suggest that even silent mutations in drug transporter genes could contribute to therapeutic failure, underscoring the complexity of resistance mechanisms in *Leishmania*, particularly in immunocompromised hosts.

Finally, non-synonymous SNPs located in coding regions are considered particularly important because they can result in amino acid changes at the protein level. Such variations may reflect functional differences that influence parasite phenotypic characteristics (Samarasinghe et al., 2018).

The first category of non-synonymous SNPs is labelled as ‘missense’ (i.e. changing the AA content), which was identified in many genes. For instance, conserved metabolic genes, such as glucose-6-phosphate isomerase (gpi), have been shown to distinguish isolates within a population and to influence pathogenicity (Zhang et al., 2014; Ceccarelli et al., 2018). In our study, gpi serves as a key genetic marker. This enzyme has historically been used for strain discrimination by multilocus enzyme electrophoresis (MLEE), and we previously incorporated it into multilocus sequence typing (MLST) analyses to investigate the genetic diversity and phylogeny of *L. infantum* in Morocco (El Mazini et al., 2023). Another missense SNP was identified in putative phosphatidylinositol 3-kinase (PI3K), a protein implicated in host–parasite interactions and host immune responses during *Leishmania* infection (Kima, 2016). A third one was detected in cysteine peptidase clan CA, family C2 (putative); these proteases play essential roles in parasite survival and proliferation within mammalian hosts and are well characterized as virulence factors as well as promising vaccine candidates in *Leishmania* species (Mottram et al., 2003).

Thirty-nine non-synonymous SNPs, labelled ‘stop-gained’, were identified across all isolates, of which seventeen occurred in hypothetical proteins. The majority stop-gain SNPs were detected in the FA23 hybrid isolate. For instance, one was identified in cysteine peptidase B (CPB), which similar to the clan CA peptidase (see above) are critical for parasite survival, proliferation, and virulence within mammalian hosts (Mottram et al., 2003). Another one was identified in heat shock protein 100 (HSP100); the expression of heat shock proteins is essential for the survival and proliferation of *Leishmania* cells in both promastigote and amastigote stages. These proteins contribute to thermo-tolerance, increased virulence, and enhanced adaptability to hostile host environments (Hombach et al., 2015). One more SNP, was found in pentamidine resistance protein 1 (PRP1); the ABC transporter PRP1 (ABCC7) has been shown to confer resistance to pentamidine in the promastigote stage of *Leishmania major* (Coelho et al., 2007).

A third category of non-synonymous SNPs is labelled as ‘stop_lost’; one such SNP was detected in putative inositol-pentakisphosphate 2-kinase (InsP5 2-kinase) that catalyzes the ATP-dependent phosphorylation of inositol pentakisphosphate (InsP5) to produce inositol hexakisphosphate (InsP6). By mediating InsP6 synthesis, this gene plays a crucial role in maintaining cellular homeostasis and regulating gene expression (González et al., 2010). Another stop_lost SNP was detected in isolate LU22 in the gene encoding for a putative microtubule associated protein involved in cytoskeletal organization (Rochlin et al., 1999),. which may allow parasites to preserve and modulate their morphology, thereby promoting adaptation to diverse environments and enhancing survival (Mule et al., 2024).

Yet another SNP category is the ‘splice_region_variant&stop_retained_variants’. One such SNP was detected the 60S ribosomal protein L32 implicated in ribosome assembly and protein translation. Ribosome-independent functions of these proteins have also been documented; the functional role that specialized ribosomes and mitoribosomes play in the response of the parasite to environmental conditions, the life cycle of the parasite, or its virulence remain unanswered fundamental questions that require further studies (Rodríguez-Almonacid et al., 2023).

The last category of SNPs are labelled ‘start_lost SNPs’. Three such SNPs were detected in hypothetical proteins and one in FA23 in a class I transcription factor A subunit 2 putative (TFIIA). Eukaryotic RNA polymerases are not capable of binding by themselves to promoters. Promoter-binding relies on transcription factors such as TFIIA, a member of the preinitiation complex that contains RNA polymerase II and six general transcription factors; TFIIA, TFIIB, TFIID, TFIIE, TFIIF, and TFIIH (Shen, 2019).

The dermotropic *L. infantum* strain FA23 isolated from a *L. tropica* endemic focus in northern Morocco showed the highest number of SNPs and occupied the most distant position in both PCA and dendrogram analyses. This genetic divergence suggest a potential hybrid origin, a hypothesis supported by comparative findings from Italy, where similarly divergent *L. infantum* isolates displayed hybrid characteristics (Bruno et al., 2024). Interestingly, a similar pattern of genomic divergence has been documented in Moroccan *L. tropica*, where two isolates clustered distantly from a core group of strains, forming a unique genomic signature distinct. from global variants (Talimi et al., 2024). Together with the hybrid-like *L. infantum* strain FA23; these findings suggest that region-specific divergence driven by geographic isolation, ecological adaptation, or hybridization may be a recurring feature of *Leishmania* evolution in Morocco.

Hybridization within the genus *Leishmania* is neither recent nor rare. The first evidence of natural hybrids dates back to 1987, with the identification of two interspecific hybrids between *L. arabica* and *L. major* isolated from animals in Saudi Arabia (Kelly et al., 1991). Subsequently, several studies have shown that genetic exchanges in *Leishmania* is both frequent and abundant (Ravel et al., 2006; Nolder et al., 2007; Rogers et al., 2014).

The detection of a potential hybrid in Morocco holds a particular significance. As highlighted by Kato et al. (2021), hybrid *Leishmania* strains may exhibit enhanced virulence, increased transmissibility, and greater adaptability compared to parental lineages. An example comes from Portugal, where natural *L. infantum/L. major* hybrids isolated from HIV co-infected patients displayed heightened vectorial competence, capable of surviving in the specific sandfly vector of *L. major* (Ravel et al., 2006; Volf and Sadlova, 2009). Such hybrids could lead to more severe clinical manifestations and broader geographic spread.

Moreover, hybridization poses a critical challenge for disease control. Genetic recombination between species may facilitate the rapid dissemination of drug-resistance traits (Ravel et al., 2006), underscoring the urgent need to investigate how these exchanges influence *Leishmania*’s transmission dynamics, pathogenicity, and ecological persistence in natural setting (Kato et al., 2021).

## 5 Conclusion

This study represents the first comprehensive genomic preliminary exploration of *Leishmania infantum* strains in Morocco, providing insights into their genetic diversity, adaptive mechanisms, and potential implications for disease pathology and control. Our findings highlight the dynamic nature of *L. infantum* genomes, characterized by widespread aneuploidy, gene copy number variations (gCNVs), and single nucleotide polymorphisms (SNPs), all of which contribute to the parasite’s ability to adapt to diverse host environments.

The detection of a potentially hybrid *L. infantum* strain (FA23) with significant SNP divergence further emphasizes the role of genetic recombination in shaping *Leishmania* diversity. This finding, supported by similar observations in *L. tropica* and hybrid strains from other regions, suggests that hybridization may be a key driver of *Leishmania* evolution in Morocco, with implications for increased virulence, transmissibility, and drug resistance.

Furthermore, the genomic heterogeneity observed in an *L. infantum* strain from an HIV-coinfected patient highlights the influence of host immune status on parasite diversity and treatment outcomes. Synonymous SNPs in drug transporter genes, though silent at the protein level, may still contribute to therapeutic failure, illustrating the complexity of resistance mechanisms in immunocompromised hosts.

As future directions and given the limited sample size in this study, expanded genomic surveillance of *Leishmania* isolates particularly from understudied regions and high-risk populations is essential. Future research should focus on (i) enlarging the scale of genomic studies to confirm strain-specific adaptations and hybrid prevalence, (ii) functional assays to elucidate the impact of CNVs and SNPs on parasite fitness, drug resistance, and host interactions, and (iii) epidemiological monitoring of hybrid strains to assess their clinical and ecological consequences.

By integrating genomic data with clinical and ecological studies, future studies will advance our understanding of *Leishmania* evolution and improve targeted interventions for this neglected but pervasive disease.

## Data Availability

The five sequenced isolates SLH16, FA23, LC_86, LU22 and LEM00 were deposed at the NCBI database and they are accessible under the following BioSample accessions: SAMN57310464, SAMN57310465, SAMN57310466, SAMN57310467, and SAMN57310468, respectively. The BioProject ID is PRJNA1454524.
